# The Path to Innovation: The Antecedent Perspective of Intellectual Capital and Organizational Character

**DOI:** 10.3389/fpsyg.2018.02445

**Published:** 2018-12-04

**Authors:** Jingyi Li, Dengke Yu

**Affiliations:** ^1^School of Economics and Management, Nanchang University, Nanchang, China; ^2^School of Management, Nanchang University, Nanchang, China

**Keywords:** organizational character, intellectual capital, technological innovation, business model innovation, structural equation modeling

## Abstract

**Purpose:** The high-speed growth of China's large-scale new economy indicates that innovation has become the most important economic growth pole. The study aims to explore the structure of the path to innovation, in which we focus on the mediating effect of organizational character.

**Design/methodology/approach:** Considering the indigenous context of China's new economy, the study divides innovation into two types: technological innovation and business model innovation. Then, we build a path model to achieve the innovation by taking intellectual capital and organizational character as antecedents. Finally, a structural equation model is built to measure the path on the basis of sample data collected via a questionnaire survey.

**Findings:** The results indicate that intellectual capital has a significant positive direct effect on technological innovation, but its direct effect on business model innovation is not significant. Organizational character not only mediates the relationship between the intellectual capital and technological innovation, but also plays a mediating role in the effect path from the intellectual capital to business model innovation. In addition, technological innovation has a positive impact on the business model innovation, and mediates the relationship between the intellectual capital and business model innovation.

**Originality/value:** The study takes intellectual capital and organizational character as the common antecedents of innovation and breaks down the content of innovation research into technological innovation and business model innovation. Thus, it establishes a new theoretical analysis framework for dual innovation research and enriches the related theories. The framework would have stronger explanatory power for revealing the innovation strategy and behavior carried out by a large number of corporate organizations in China and the boom of new economy. Furthermore, it would lead enterprises to organize innovation activities more effectively and improve their innovation performances.

## Introduction

At present, China is experiencing an unprecedented high-speed development. The force formed by the wide utilization of internet in e-commerce and the increasingly raised high-tech enterprises is driving the vigorous development of China's new economy. The mechanism underlying the force is innovation, including technological innovation and business model innovation (Sagar and Zwaan, 2006; Gambardella and McGahan, 2010). On the one hand, due to the technological innovation, technological breakthroughs and industrial upgrading have enabled many large enterprises in China to establish their core competencies based on high technology, such as, Huawei (Lee et al., 2016). On the other hand, due to the business model innovation, the reform of corporate profit model and the reshaping of industrial value chain have enabled many small and medium-sized enterprises in China, for example Didi, to gain temporary competitive advantage relying on the differentiation strategy in marketing. Even some leaders (e.g., Alibaba and Tencent) in business model innovation can create a bran-new economic-society-ecosystem that may affect the progress of human civilization (Haenninen et al., 2018). In a word, innovation become the fundamental driver of China's economy. The exploration of the path to innovation in Chinese corporate organizations has important theoretical value and is also of great significance to the innovative development of corporate organizations.

In the background of knowledge economy, intellectual capital is the basic resource of economy (Tseng and Goo, 2005) and the core source of sustainable competitive advantage for corporate organizations (Kang and Snell, 2009; Youndt et al., 2010). The researchers in the study field of technological innovation theory always believe that technological innovation plays an important intermediary role in the acting path from intellectual capital to sustainable competitive advantage (Engelman et al., 2017). Numerous researches have demonstrated the close relationship between intellectual capital and technological innovation (Costa and Dorrego, 2014). For example, some studies concentrate on the exploration of the relationship between organizational knowledge and new product development (Yang and Rui, 2009), the description of innovation process from the perspective of knowledge management (Castro et al., 2013; Cabrilo and Dahms, 2018), and the formation mechanism of innovative organization from the perspective of knowledge-creating procedures (Buenechea Elberdin et al., 2018). Therefore, the design that takes intellectual capital as an antecedent to build the path to technological innovation logically makes sense.

We should naturally consider the next question: what is the antecedent of the business model innovation? According to the observation of Chinese companies, it has been found that the methods most companies used to achieve business model innovation are mainly reflected in the adoption of new logistic channels, the introduction of new payment modes, and the improvement of consumer experience as well as the creation of new profit models, etc. The target of employing these new methods is to differentiate their products or services from competitors, thus helping them to build differentiation competitive advantages. According to the competitive strategy theory, the business strategy is summarized into three types: cost leadership strategy, differentiation strategy, and focus strategy (Porter, 1980). It can be seen that the differentiation is important to the survival and development of corporate organizations. Resnick (2003) ulteriorly pointed out that both core competitiveness and differentiation competitiveness constitute an organization's competitiveness together; the sources of core competitiveness are knowledge and technology, while the differentiation competitiveness can be driven by organizational character. According to the study of Moore (2005), organizational character is a relatively broad concept, which includes the diversity of corporate strategy, the specificity of organizational culture, and the heterogeneity of corporate products as well as the integration of employees' character. A corporate organization can utilize the differentiation of organizational character to continuously break the existing market competition structure and create opportunities for organizational development. Therefore, this study sets the organizational character as the antecedent of business model innovation and makes it join the path to innovation for further discussion.

In addition to the above two paths, what kinds of relationship exist among intellectual capital, organizational character, and technological innovation as well as business model innovation? First, according to the practical observations, we find that the organizations of which the business model innovations lack core technical support but only be differentiated for differentiation cannot survive long in market competition. For example, Didi, which engages in the development of one-stop travel platform, has flourished in the past few years because of its shared business model. However, it began to face many challenges in sustainable development in recent period because of the lack of supervision technology and distributed management technology, when the volume of its passenger flow has a sustained growth. Hence, only the ones which have combined the two kinds of innovation and make the changes of business model supported by technological transformation and product upgrading can sustain for a long time. Making technological innovation lead business model innovation has become a way for Chinese companies to keep success.

Second, intellectual capital do affect the formation of organizational character. With the creation and accumulation of intellectual capital, corporate organizations can gradually change their strategic positions, decision-making styles and behavior styles (Peppard and Rylander, 2001; Marr et al., 2003; Tseng et al., 2013), thus continuously and unconsciously changing their organizational characters. For instance, Huawei in the pioneering period was famous for its “wolf spirit,” which encourages itself to win the competition based on the actions characterized by struggle, barbarity and greed. However, its organizational image becomes to be knowledgeable, creative and socially responsible when it grows to be a world-renowned enterprise. The establishment of the new organizational image is benefited by its continuous high-intensive R&D inputs and continuous high productivity of patent.

Third, every type of business model innovation is derived from creativity, which is related to the talent and knowledge (Hunter et al., 2008). Moreover, the realization of business model innovation requires the support of human capital, social capital, and organizational capital (Ujwary-Gil, 2017). Consequently, intellectual capital should also have a driving effect on business model innovation. For example, Alibaba develops into one of the best famous enterprises in China on account of its leading business model and particular business actions, e.g., Double Eleven and Double Twelve. We know the fact that the continuous successes of Alibaba's business actions are profited from its powerful data handling capability, integrative resource allocation network and effective teamwork on business model design.

Finally, technological innovation involves many aspects of decision-making such as innovation direction choices, innovation investments and innovative applications. These decisions are closely related to organizational strategy, organizational culture, and organizational structure (Lemon and Sahota, 2004; Sarros et al., 2008). In other words, organizational character may have a significant impact on technological innovation. For instance, a well-known Chinese enterprise, Giant Group, developed rapidly from 1991 to 1995 and suddenly closed down in 1996 because of capital chain rupture. In fact, the deep-rooted reason is that it continuously transferred the profits of main businesses to establish the highest building in Zhuhai city instead of using the money to support R&D activities and sustainable growth. Hence, the failure of Giant Group can be more attributable to its vanity, which misled its technological innovation decision.

Based on the above considerations, we employ the intellectual capital as an antecedent variable and the organizational character as a mediating variable to develop the path to technological innovation and business model innovation. Under the circumstance of China's new economy, this path model could have a strong explanatory power for the innovative development mechanism of a corporate organization. Therefore, this study has strong contextualized characteristics in terms of model construction and sample selection. That is, this article plans to focus on the report of research results in Chinese context, but somehow, its research conclusions would be applicable to all corporate organizations under new economy circumstances and should be able to teach lessons to organizations under other cultural backgrounds for innovative development.

Compared with previous studies, the research may make the following theoretical contributions. First, the new dual innovation model consisting of technological innovation and business model innovation broadens the existing concepts of dual innovation and helps to explore a new way to innovation. Second, we bridge the relationship between intellectual capital and innovation firstly by organizational character. This would lead more researchers to consider and appreciate the functions of organizational character. Third, we provide a quantitative method to measure and analyze the organizational character. In addition, the contribution of this research covers that it provides a better explanation for the success and failure of many Chinese enterprises' innovations in current environment which characterized by high competition, quick change and diversified development.

We organize the remainder of the paper as follows. In section Theory and Hypotheses, we develop the research hypotheses on the basis of theoretical analysis and literature review. In section Research Design, we conduct the research design including measurement, data collection and methods. Then, we present research results and generate path model in section Results and discuss the results and analyze their theoretical values and management implications in section Discussion and Implications. Finally, we summarize the conclusions and limitations, and point out the research potentials in the future in section Conclusions, limitations, and Future Research.

## Theory and Hypotheses

Since the innovation theory was proposed by Schumpeter in the early twentieth century, technological innovation has been widely concerned (Li and Atuahene-Gima, 2001; Casadesus Masanell and Zhu, 2013). Technological innovation conceptually involves the creation of product and process innovations (Camisón and Villar-López, 2014). In contrast, business model innovation is a type of innovation that has only begun to be specially concerned about after the twenty-first century (Teece, 2010). It considers the business model instead of products or processes as the center of innovation (Baden-Fuller and Haefliger, 2013). According to the practical experiences in current China, the drivers that trigger business model innovation are the large-scale application of internet technology in e-commercial field and the booming digital economy. In the era of new economy, business model describes the basic principles of value creation, value proposition and value realization (Clauss, 2017). It shapes the core logic of organizational value increment and becomes the core content of organizational operation strategy. Correspondingly, business model innovation has become an important competitive advantage source alongside the technological innovation by leading the innovation of organizational value realization model (Demil and Lecocq, 2011). Giving an overall consideration to both the analysis of innovation theory and the observations from Chinese practices, this research aims to explore the path to innovation by two-fold focus on the technological innovation and business model innovation.

### Intellectual Capital and Technological Innovation

Intellectual capital is the knowledge that a corporate organization has and uses for creating value (Stewart, 1997), including employee skills, customer loyalty, and all knowledge resources embedded in organizational culture, systems and processes. There are many kinds of viewpoint about the composition of intellectual capital, such as two-, three-, and four-element theories (Curado, 2008; Martín-De-Castro et al., 2011). The widely accepted viewpoint is the three-element theory which decomposes intellectual capital into three dimensions: human capital, social capital, and organization capital (Subramaniam and Youndt, 2005).

Human capital promotes technological innovation by reducing the risk of technological innovation. Human capital refers to the knowledge, experience, and ability of all individuals in a corporate organization (Subramaniam and Youndt, 2005). It has been demonstrated by many previous researchers. On the one hand, employees with solid professional knowledge and rich technical experience would be more accurate when they grasp the potential direction of innovation and can control possible errors in the operation of technological innovation (Thornhill, 2006). They can make technological innovation more efficient and with lower risk, thus causing it more active. On the other hand, the knowledge structure and professionalism of top managers can influence decision-making (McNamara et al., 2002). The level of education of top managers would also lead different decisions about technological innovation (Kimberly and Evanisko, 1981). The education level can represent the knowledge ability of top managers to some extent (Wally and Baum, 1994). High knowledge ability could help top managers make right decisions and lead the success to technological innovation.

Social capital enables organizations to quickly perceive customer demands and market changes, thus forming a resource delivery network to share and provide resources for technological innovation. Selden and Macmillan (2006) believed that the cornerstone of one's successful innovation is to understand its customers and markets. When the relationship between a company and its customers is harmonious, it can promptly perceive market changes through customer feedback and then adjust the direction of technological innovation. Besides, the social capital theory also emphasizes the relationship between a corporate organization and its suppliers. In order to better realize the ideas and arrangements for technological innovation, organizations need to rely on the cooperation and collaboration of suppliers (Lau et al., 2010; Joshi, 2017). Moreover, in some cases, the cooperation with suppliers may explore and provide with appropriate approaches for organization's technological innovation (Pulles et al., 2014). In addition, organizations can also actively develop relationships with other external organizations (e.g., government, university, and research institute) and establish social networks centered on open innovation (Stam and Elfring, 2008; Diez-Vial and Montoro-Sanchez, 2014). Social networks enhance resource exchange, energy flow and value conversion between organizations and the environment, and further provide platform and resource supports for collaborative innovation, thereby enhancing organizations' technological innovation capabilities.

Organization capital can improve the efficiency of internal communication and operation in organizations, thus ensuring the full realization of technological innovation. Organization capital is the intangible asset embedded in an organization. It is usually represented by strategic culture, organizational systems, management rules, databases, information platforms, and so on (Subramaniam and Youndt, 2005). The high level of organization capital means that the corporate organization is relatively mature and perfect at the management level, and the communication and operation efficiency as well as resource flow efficiency would be relatively high (Qian et al., 2014). In the process of technological innovation, organizations need to invoke a large number of innovative resources, and the organizations with rich accumulation of organization capital thereby would have advantages in the efficiency of technological innovation (Chen and Inklaar, 2016). For example, in China, Huawei is famous for its patent development. Its technological innovation capability far ahead of its competitors has benefited greatly from the Integrated Product Development (IPD) process introduced from IBM. Through the implementation of IPD, Huawei gradually established a world-class R&D management system, formed excellent R&D capabilities, and made significant contributions to the core competitiveness (Lee et al., 2016).

In summary, human capital, social capital, and organization capital all play an active role in technological innovation activities. Hence, intellectual capital can be identified as an important antecedent variable of technological innovation. We therefore put forward the following hypothesis:

*H1*. Intellectual capital has a significant positive impact on technological innovation.

### Intellectual Capital and Business Model Innovation

Similar to the effects on technological innovation, human capital, social capital and organization capital would also play an important role in promoting business model innovation. First, the driving mechanism of human capital for business model innovation is mainly reflected in the influence of top managers' leadership, since the leaders of business organizations are usually also the designers of business model (Zott and Amit, 2007). A leader's strategic foresight, judgment ability, personality preference and behavior model would have profound impacts on organization's decision-making in business model innovation (Martins et al., 2015; Velu and Jacob, 2016). Business model innovation requires some specific leaderships (Svejenova et al., 2010) such as cognitive ability to the market, conviction (Aspara et al., 2011) and creativity (Svejenova et al., 2010). Leaders may promote the initial business model design through intention, but they would make continuous decisions on whether to maintain the status quo or to imitate competitors' business models in the market (Casadesus Masanell and Zhu, 2013).

Second, the intra-industry learning and cross-industry learning induced by social capital make business model innovation possible and diverse. Intra-industry learning helps organizations find a new way to coordinately create value across their boundaries accompany with upstream and downstream firms. Organizations can discover new value propositions, competitive positioning, and new sources of value through learning from their existing customers, competitors, and suppliers (Kang et al., 2007). Some studies could support such viewpoint. For example, Chesbrough (2010) proposed that the deep understanding of potential customer needs is an important driving force for reshaping the value creation system. Zott and Amit (2008) also believed that the business model of competitors is an important template for organizations to redesign their business models. In addition, cross-industry learning can help organizations adopt strategies that are different from their peers and change their business paths without features (Atuahenegima and Murray, 2007; Stam and Elfring, 2008; Bock et al., 2012). This would help organizations develop new ways of value creation, explore new profit points, and lead old business model to achieve great-leap-forward innovation.

Finally, organization capital can raise the value creation and process efficiency of business model. Several existing researches have shown that organizational processes contribute to corporate organizations when they coordinate the strategy, structure, culture, and routine work for efficient operations (Garicano and Wu, 2012; Keramati and Shapouri, 2016). Advanced information systems and technology platforms are conducive to information collection and knowledge sharing (Yang et al., 2012), so they could aid decision-making, promote collaboration, reduce operational costs, and also increase operating efficiency (Han and Li, 2015). Furthermore, unique procedures or processes that perform tasks and activities are potential sources of innovation performance (Isaksen and Nilsson, 2013). The organizations with poor procedures and systems could not be able to realize their potential, while the others with abundant organization capital are efficient in value creation activities (Widener, 2006). We thus put forward the following hypothesis:

*H2*. Intellectual capital has a significant positive impact on business model innovation.

### The Mediating Role of Organizational Character

#### Intellectual Capital, Organizational Character, and Innovation

Similar to individuals, groups may also exhibit relatively stable trait preferences and behavior patterns, that is, the emergence of group characters. Organizational character is the concrete manifestation of group personality (Yu et al., 2018). The personality trait is the fundamental factor that determines an individual's decision-making and behavior (Schneider et al., 1995). Correspondingly, up to the organizational level, organizational character should be able to explain organizational behavior and predict organizational performance (Yu et al., 2018). Innovation, as a strategic behavior that dictates the evolutionary direction of an organization, would naturally be greatly affected by the organizational character. Some practical examples would support this deduction. For instance, risk-taking organizations often prefer to choose radical innovation strategy (Cai et al., 2015), while robust organizations are usually biased toward choosing incremental innovation strategy (McDermott and O'Connor, 2002); open organizations always tend to employ collaborative innovation strategy (Chesbrough, 2003), while conservative organizations generally like to adopt internal innovation strategy (Felin and Zenger, 2014), and; high-tech and knowledge-intensive organizations mostly tend to design technological innovation strategy, while organizations that are good at learning, flexibility and adaptability are inclined to rely on business model innovation strategy, etc.

According to the Attraction-Selection-Attrition (ASA) theory constructed by Schneider et al. (1995), the formation process of organizational character can be divided into three stages. First, the top management team plays a key role in organization design and creation (Giberson et al., 2005). The personality and behavior of entrepreneurs would inevitably have a decisive influence on the initial-stage organization's strategy, positioning and culture (Baptista et al., 2014; Urbano et al., 2017). Next, along with the development and growth of the organization, the number of employee gradually increases. Individuals with different characters increasingly achieve assimilation by adapting, attracting, selecting, and discarding their own personalities. At length, the assimilated character of individual employees re-aggregates and shows a common trait at the group level, namely, organizational character.

From the above description of the formation process of organizational character, it can be seen that the role of intellectual capital runs through the entire process. The birth of organizational character depends on the lead of human capital such as top management team and employees. Individual character assimilation is achieved through the exchange of employees and interaction within the organization (Schneider et al., 1998). These are all driven by relational capital. The process of individual character assimilation also needs to be supported by the organizational management philosophy and system architecture (Newman, 1953), which includes organizational structure, management system, and database platform and so on. Obviously, those can be summarized in the category of organization capital.

To sum up, organizational character is the influencing factor of innovation, and the formation of organizational character is driven by intellectual capital. We propose the following hypothesis since we have analyzed the positive impact of intellectual capital on innovation.

*H3*. The organizational character mediates the positive relationship between intellectual capital and innovation.

#### Intellectual Capital, Organizational Character, and Technological Innovation

In general environment of knowledge economy, with the gradual accumulation of intellectual capital, organizations always choose to create a culture that respects knowledge talents, thinks highly of knowledge resources, and aims to realize knowledge performance (Hsu and Sabherwal, 2012; Asiaei and Jusoh, 2015). Because of the edification of this kind of knowledge-based culture, organizational characters would be also full of knowledge characteristics. A knowledge-based organization shaped by knowledge-based culture always expects to take full advantage of its intellectual capital efficiently. In terms of specific behaviors, they are willing to share, disseminate, innovate and utilize knowledge resources (Alawi et al., 2007; Ajmal and Koskinen, 2008; Wu and Lee, 2016) in order to support technological innovation and knowledge transfer. With the evolution of innovation mode, it is now entering the stage of open innovation (Chesbrough, 2003). With the trend of open innovation, organizational personality trait would be more vivid in openness, knowledge, and sharing (Westergren and Holmstrom, 2012). Organizations tend to cross their boundaries, accelerate internal innovation purposefully through the inflow and outflow of knowledge, and develop the two independent processes of internal innovation and external application market (Chesbrough, 2006). The knowledge-sharing organizational character and social capital would jointly drive the knowledge exchange within and between organizations in order to promote resource exchange and utilization (Shao et al., 2015). Such behavior would further raise organizations' resources and capabilities and strengthen collaborative technological innovation. Therefore, we propose the following hypothesis:

*H3a*. The organizational character mediates the positive relationship between intellectual capital and technological innovation.

#### Intellectual Capital, Organizational Character, and Business Model Innovation

Intellectual capital, especially the one obtained from external exchange and learning process, would gradually change the organization character, so as to incorporate the organization character into the external environment and enhance the organization's adaptation to the environment. Furthermore, both the accumulation of intellectual capital and the evolution of organizational character would increase the organization's perception of market changes (Han and Li, 2015), enhance its own strategic adjustment (Bratianu and Orzea, 2013), and strengthen the ability to improve upstream and downstream integration of suppliers and customers (Zhang et al., 2018). As a result, they help organizations to explore new ways of value creation and realize business model innovation. Some practical observations showed that the more intellectual capital an organization accumulates, the greater force that emphasizes knowledge exchange and growth it may form (Huang et al., 2010), and it is more able to keep an open and inclusive attitude to face external shocks and correspondingly make positive adjustments (Han and Li, 2015). In a word, such organization can acquire more market opportunities. When opportunities arise, the organization with rich intellectual capital can quickly identify and exploit them (Ramos-Rodriguez et al., 2011). In addition, organizations would continuously design, test, adjust, and improve their initial business models through trial and error learning (Morris et al., 2005; Sosna et al., 2010). They can create new business models by identifying, optimizing, adapting, revising, and reshaping the old ones (Morris et al., 2005). Through the above mentioned approaches, the innovation of business model is achieved. Thus, we propose the following hypothesis:

*H3b*. The organizational character mediates the positive relationship between intellectual capital and business model innovation.

### Technological Innovation and Business Model Innovation

There is no value in technology itself, so its potential economic value must be achieved through business activities (Elia et al., 2017). Business model innovation is a process by which an organization could establish its business logic and relate the technology to the underlying economic value it entails. Each organization should establish a business model that matches its core technologies for achieving sustainable economic success (Chesbrough, 2006; Wei et al., 2014). Technological progress brings new opportunities for organizations, partners, and customers to coordinately upgrade value chain and innovate business model (Mendelson, 2000; Geoffrion and Krishnan, 2003). For example, the booming sharing economy in China, which reflects as various business model innovations in emerging industries, is fundamentally supported by the development and wide application of mobile internet technology. All in all, technological innovation can catalyze business model innovation. Thus, we propose the following hypothesis:

*H4*. Technological innovation has a significant positive effect on business model innovation.

**Figure 1:**
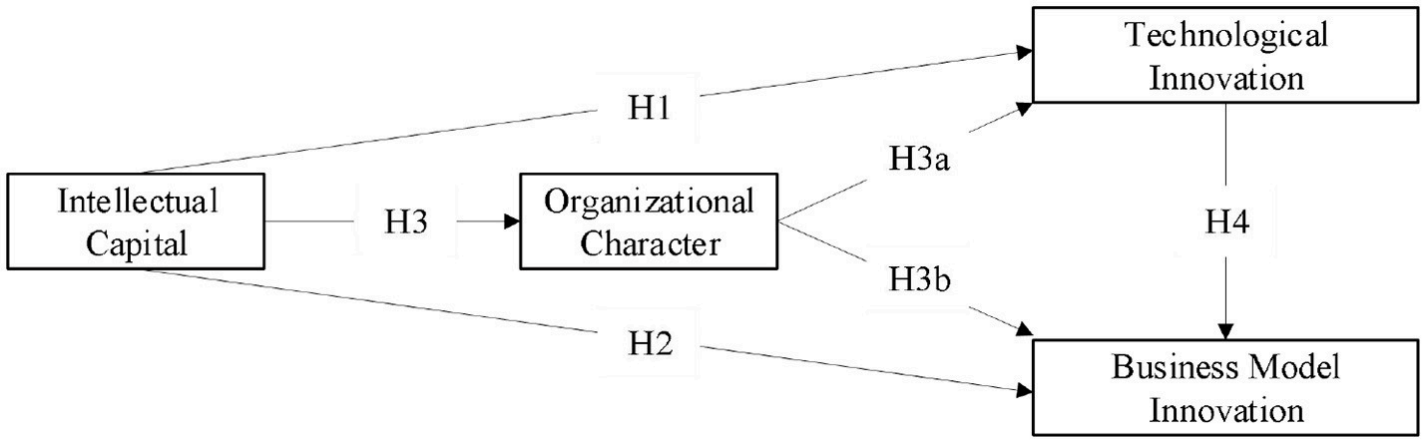
Research framework.

Based on the above research hypotheses, the research framework of this study is constructed as shown in Figure 1.

## Research Design

### Measures

#### Latent Variables

First, intellectual capital. According to the intellectual capital theory, we measure intellectual capital from three dimensions: human capital, social capital, and organization capital (Subramaniam and Youndt, 2005). Drawing on the intellectual capital scale developed by Subramaniam and Youndt (2005), we screen out items that fit the context of Chinese corporate organizations and merge them into six new items. Each dimension is measured with two items.

Second, organizational character. In the past, the measurement of organizational character always drew lessons from individual personality scales. For example, Chun and Davies (2006) developed an organizational character measurement scale by means of adapting the Big Five personality scale in the field of personality psychology. In their scale, the organizational character is divided into five dimensions to measure, namely, agreeableness, enterprise, competence, chic, and ruthlessness. According to the author's observation of Chinese corporate culture and social culture, it is believed that the dimension of ruthlessness is not suitable for describing most of Chinese organizations. Therefore, this research measures organizational character from the first four dimensions, and each dimension is considered by two items.

Third, technological innovation. In broad sense, technological innovation includes the complete process of developing new technologies and products, bringing them to market and successfully commercializing them (Freeman and Soete, 1997). In order to distinguish it from business model innovation, this study adopts the definition that “technological innovation involves product and process innovations, while non-technological innovation involves marketing and organizational innovations” (Camisón and Villar-López, 2014). Thus, we observe the technological innovation from two dimensions: product innovation and process innovation. Drawing on the scale designed by Camisón and Villar-López (2014) and selecting and adapting the items based on Chinese contexts, we finally choose six items to measure the technological innovation. Each dimension is measured by three items.

Finally, business model innovation. In fact, the definition of business model has not yet reached a consensus (Zott et al., 2011). From the perspective of corporate value, this study defines the business model innovation as the activities that mainly aim to innovate or adopt new value creation models for organizations (Clauss, 2017). The measurement of business model innovation is adapted from the novelty-centered business model design scale developed by Zott and Amit (2007). After selection, we retain 6 items from the original 11 items.

#### Control Variable

First, environmental uncertainty. Previous studies have shown that the environmental uncertainty can have a significant impact on business model innovation (Camisón and Villar-López, 2014; Huang et al., 2017). For most of the time, organizations continue to innovate business models (Desarbo et al., 2005) to better fit the changes of market demand in external environments. Accordingly, we set the environmental uncertainty as a control variable for business model innovation. Based on the studies of environmental characteristics, e.g., Dess and Beard (1984), two items are designed to measure the environmental uncertainty.

Second, hi-tech enterprise certification. Hi-tech enterprise certification is a recognized act for funding the potential high-tech companies that meet the national or local requirements on technological innovation. Chinese enterprises which have successfully obtained hi-tech enterprise certification may receive a lot of supports such as tax deduction and deduction on R&D expense from the national and local governments. All in all, the hi-tech enterprise certification would have a significant positive impact on the technological innovation, so it needs to be set as a control variable.

In addition, given that the ownership structure, staff size, age, industry also affect business model innovation and technological innovation (Camisónzornoza et al., 2004; Damanpour and Aravind, 2006), we set them as control variables as well.

#### Measure Items

With regard to these variables, namely intellectual capital, organizational character, technological innovation, business model innovation, and environmental uncertainty, their measurement items are selected and adapted from the mature scales in previous literature. The whole process of selection and adaptation is discussed by the entire research team. The process follows the following principles: first, we delete the items that are not applicable to Chinese enterprises; second, we adjust the items of which the language expression does not conform to the language habits of Chinese; third, we try to summarize the items with similar meanings in Chinese in order to balance the item volume of different latent variables. These selected and adapted measurement items form a new scale together. The new scale has also been confirmed by several experts who have long-term research experience in the field of organizational management.

As most of the original scales were developed in English and applicable to English-speaking countries such as Europe and the United States, a back translation process is adopted to edit the survey. We invite two students majored in both English and management to translate our scale. One student translates the selected scale into Chinese first and then the other one translates the Chinese version into English to meet the requirements of content validity (Lawshe, 1975). After several rounds of back translation process, the scale in Chinese version is confirmed. The new scale would be more suitable for observing corporate organizations in Chinese context semantically and easily understood by the employees of Chinese enterprises. The items of the above five variables are designed in the form of Likert's five-point scale, ranging from 1 (“strongly disagree”) to 5 (“strongly agree”).

In the end, we generate a questionnaire with 36 items, in which six items are set for business model innovation, six items for technological innovation, six items for intellectual capital, eight items for organizational character, two items of environmental uncertainty, and five items reflect other control variables and three items are used to identify the identity of respondents. Variables and items are shown in Table AI (see Appendix in the Supplementary Material).

### Data Collection

We upload the edited questionnaire to Sojump, which provides a professional survey service, and entrust it to collect data. In China, Sojump is a large professional online survey service platform (http://www.sojump.com). So far, the platform has provided more than 1.6 billion questionnaires for 24.67 million users. More than 100 million people participated in filling the questionnaire. In addition, 113 of the world's top 500 institutions have used sample services provided by Sojump. Many Chinese scholars have also sought help from it during their academic research processes (Che and Cao, 2014; Rui, 2017).

The questionnaire of this study was issued from April 4 to April 9, 2018. Through Sojump, 580 questionnaires were distributed and 366 questionnaires were returned (recovery rate is 63.10%), of which 214 are valid questionnaires (effective recovery rate is 36.89%). According to the suggestion of Fritz and Mackinnon (2007), the sample size can meet the requirements of the estimation of the parameters in our model.

This study uses the platform's sample database for random sampling, which effectively reduces the selection bias of the respondents. According to the identification of IP address when respondents complete questionnaires online, we find that the respondents are from 28 different provinces or municipalities in mainland China, which is divided into 31 provinces or municipalities in total. According to our statistics, the respondents are mostly from eastern coastal areas, and the ones from western or northeastern regions are relatively few. This is basically consistent with the regional distribution of Chinese economic development. Through statistics on the identities of the respondents and some control variables (see Table 1), it can be seen that the education background of most respondents is at or above the undergraduate level, indicating that they should understand the questionnaire well; their service year is long enough to have a full understanding of their respective organization's situation, and; they are mainly manager and technician, with which positions the employees themselves are the most important human resources and should answer the questionnaire clearly. Moreover, the respondents are evenly distributed across different industries, sizes and ages. The number of respondents from high-tech certified enterprises and non-certified enterprises is with little difference. The distribution of the ownership structure of the respondents is in line with Chinese contemporary mixed ownership economy. These statistics show that the sample of this survey has a certain degree of representation, and the respondents' answers can be trusted to some extent.

**Table 1:** Distribution of the respondents.

**Variables**	**Options**	**Distribution (%)**	**Variables**	**Options**	**Distribution (%)**
Educational background	Junior college or below	16.80	Industry	Primary industry	25.23
	Undergraduate degree	77.60		Secondary industry	37.38
	Graduate degree	5.60		Tertiary industry	37.38
Service year	Less than 1 year	4.70	Age	Less than 1 year	0.50
	1–3 years	30.40		1-3 years	3.74
	4–10 years	45.30		4–10 years	36.45
	More than 10 years	19.60		More than 10 years	59.35
Current job	Manager	39.70	Ownership structure	State-owned enterprise	20.56
	Technician	41.10		Foreign-capital enterprise	10.28
	Production worker	4.70		Joint-investment enterprise	9.35
	Salesman	10.70		Private enterprise	57.94
	Other	3.70		Other	1.87
Staff size	10 or less	1.40	Hi-tech enterprise certification	Yes	54.67
	10–99	30.37		
	100–300	37.85		No	45.33
	More than 300	30.37		

### Methods

We test our model hypothesis by building a structural equation model, which can simultaneously estimate the relationships between multiple latent variables that contain measurement errors. It is superior to the regression analysis method in reliability. We use Mplus 7.0 to analyze the latent variable structural equation model. Significance tests for the mediating effects are based on maximum likelihood estimation results and bias-corrected confidence intervals (CIs) derived from 1,000 bootstrapped samples. As for the reliability and validity tests, we use SPSS 19.0 to complete them.

## Results

### Descriptive Statistics

The descriptive statistical analysis results of each item are shown in Table 2. The parameter estimation method adopted in this study is Maximum Likelihood Estimation (ML), which requires the data distribution to be standard normal distribution. However, Finney and DiStefano (2006) pointed out that when the kurtosis coefficient is less than 7 and the skewness coefficient is less than 2, the non-strict standard normal distribution of sample data does not have a sufficient impact on the ML estimation. Table 2 shows that the data for each measurement item is a non-strict normal distribution, but the kurtosis coefficients are between −0.843 and 1.927, and the skewness coefficients are between −1.229 and 0.052, which are within the allowable range of the ML estimation method. Generally speaking, the structural equation model based on the ML method is suitable to this research.

**Table 2:** Descriptive statistics of latent variables.

**Latent variables**	**Items**	**Mean**	**Standard error**	**Kurtosis**	**Skewness**
Intellectual capital (IC)	IC1	3.893	0.879	1.927	−1.129
	IC2	3.645	0.912	−0.179	−0.287
	IC3	3.748	0.915	−0.337	−0.331
	IC4	3.682	0.935	−0.214	−0.402
	IC5	3.009	0.828	−0.806	0.083
	IC6	3.748	0.935	0.673	−0.796
Organizational character (OC)	OC1	3.780	0.941	−0.313	−0.468
	OC2	3.953	0.913	0.195	−0.729
	OC3	3.850	0.848	−0.610	−0.268
	OC4	3.229	0.929	−0.242	−0.046
	OC5	3.701	0.971	0.388	−0.704
	OC6	3.785	0.935	0.555	−0.777
	OC7	3.579	1.007	−0.454	−0.247
	OC8	3.696	0.933	0.026	−0.480
Technological innovation (TI)	TI1	3.350	0.895	−0.388	−0.157
	TI2	3.612	0.880	−0.180	−0.326
	TI3	3.822	0.972	0.302	−0.751
	TI4	3.262	0.928	−0.126	−0.011
	TI5	3.402	0.972	−0.573	−0.200
	TI6	3.229	0.914	−0.347	0.052
Business model innovation (BM)	BM1	3.696	0.779	0.188	−0.373
	BM2	3.402	1.029	−0.786	−0.086
	BM3	3.332	0.996	−0.431	−0.159
	BM4	3.561	1.045	−0.285	−0.536
	BM5	3.182	1.043	−0.612	−0.046
	BM6	3.565	0.868	−0.213	−0.160

### Reliability, Validity, and Common Method Bias

#### Reliability

Cronbach's α coefficient (Cronbach, 1951) is the most commonly used indicator to exam the consistency of a construct's measure items. It is generally recommended that the Cronbach's α coefficient should be above 0.7. In this study, the Cronbach's α coefficients for each variable are above 0.7 (see Table 3), indicating that the variables have great internal consistency among the items. Given that some scholars believe that the Cronbach's α coefficient does not provide a good estimate of reliability (Green and Yang, 2009; Sijtsma, 2009), the study intends to use the composite reliability (CR) to test the internal consistency of the scale again. The CR (Raykov and Grayson, 2003) is a reliability index for latent variable that is commonly used in structural equation model. In general, it is considered that the scale has good internal consistency as long as CR is above 0.6 (Bagozzi and Yi, 1988). All CR values in this study are well above 0.7 (see Table 3). In conclusion, the two methods of internal consistency test show that our scale has an appropriate reliability.

**Table 3:** Reliability and validity test.

**Variables**	**Item**	**Factor loading**	**Cronbach's α**	**CR**	**AVE**
Intellectual capital (IC)	IC1	0.830	0.727	0.857	0.501
	IC2	0.643		
	IC3	0.672		
	IC4	0.673		
	IC5	0.691		
	IC6	0.723		
Organizational character (OC)	OC1	0.831	0.766	0.891	0.508
	OC2	0.702		
	OC3	0.639		
	OC4	0.669		
	OC5	0.819		
	OC6	0.636		
	OC7	0.675		
	OC8	0.704		
Technological innovation (TI)	TI1	0.849	0.779	0.870	0.529
	TI2	0.683		
	TI3	0.810		
	TI4	0.712		
	TI5	0.640		
	TI6	0.644		
Business model innovation (BM)	BM1	0.907	0.750	0.876	0.546
	BM2	0.645		
	BM3	0.697		
	BM4	0.779		
	BM5	0.645		
	BM6	0.747		
Environmental uncertainty (EU)	EU1	0.621	0.720	0.749	0.607
	EU2	0.915		

#### Validity

The validity test includes three aspects, namely convergence validity, construct validity, and discriminant validity. At first, the average variance extraction (AVE) provides an assessment of the convergence validity. The AVE of all variables in this study reach the recommended value 0.5 (see Table 3; Fornell and Larcker, 1981). Thus, each construct in the scale has a high degree of convergence validity. Secondly, construct validity refers to the ability of inferring or measuring abstract constructs. It is usually proved by factor analysis. We calculate KMO value and make Bartlett's spherical test for each variable at first. The KMO value of each variable is higher than 0.6 recommended by Kaiser and Rice (1974), while the significance of Bartlett's spherical test values is 0.000, indicating that the scale is suitable to factor analysis. Then, confirmatory factor analysis (CFA) is used to measure the standardized coefficients of all factors. The standardized coefficients are all above 0.5 and significant at the *p* < 0.001 level, indicating that the construct validity meets the standard. The last aspect of validity is discriminant validity. It refers to the distinguishability of different constructs. To satisfy the requirement of discriminant validity, the square root of a construct's AVE must be greater than the correlations between the construct and the other ones in the model. Drawn from Table 4, the diagonal data (square root values of AVE) are all higher than their corresponding non-diagonal data (correlation coefficients). Therefore, the discriminant validity of the measurement is acceptable. According to the above results, the convergence validity, construct validity, and discriminant validity in this study are all adequate.

**Table 4:** Discriminant validity.

**Variables**	**IC**	**OC**	**TI**	**BM**	**EU**
Intellectual capital (IC)	**0.708**			
Organizational character (OC)	0.545	**0.713**		
Technological innovation (TI)	0.393	0.380	**0.728**	
Business model innovation (BM)	0.389	0.465	0.452	**0.749**
Environmental uncertainty (EU)	0.241	0.131	0.095	0.195	**0.779**

*The bold data are the square root values of AVE; the non-bold data are the correlation coefficients between the variables*.

#### Common Method Bias

In order to reduce common method bias, we adopt both procedural methods and statistical techniques. Regarding procedural methods, Podsakoff et al. (2003) proposed that “protecting respondent anonymity and reducing evaluation apprehension” can reduce common method bias. When we were collecting data, we assured the potential respondents that the data would be anonymous and only for academic research. All respondents participated in our research voluntarily. Due to the limitation of study conditions, the procedural methods we take may not completely eliminate the bias, so we need to add statistical techniques to improve our study. According to the recommendations of Guide and Ketokivi (2015) and research design of Jia et al. (2018), we used Harman's single factor test to evaluate the level of common method bias. In this research, all the items of latent variables were subjected to a factor analysis. The explained variance of the first factor among the five factor values with initial eigenvalues larger than 1 is 27.01%, less than the commended 40%. Thus, the common method bias should not be a significantly concerned problem in this study.

### Path Analysis

According to the research framework in Figure 1, we use Mplus 7.0 to perform latent variable path analysis and get the path coefficients to test the significant effects among variables. The model fitting results of this study is χ^2^ = 1101.467, *df* = 481, χ^2^/*df* = 2.29 (the recommended standard is less than 3), RMSEA = 0.078 (the recommended standard is less than 0.8), CFI = 0.926 (the recommended standard is more than 0.9), TLI = 0.998 (the recommended standard is more than 0.9), SRMR = 0.041 (the recommended standard is less than 0.05). All indicators meet the requirements, and the degree of model fitting is thus acceptable.

Table 5 reveals the standardized path coefficients of the relationships between the latent variables. After controlling the high-tech enterprise certification, ownership structure, staff size, age and industry, intellectual capital (IC) has a significant positive effect on technological innovation (TI) (β = 0.264, *p* = 0.000). Thus, H1 is supported. However, after controlling the environmental uncertainty, ownership structure, staff size, age and industry, the positive impact of IC on business model innovation (BM) is not sufficiently significant (β = 0.088, *p* = 0.078). Thus, H2 is rejected. In addition, TI has a positive effect on BM (β = 0.310, *p* = 0.000), so the result supports the hypothesis H4. Finally, the relationship between IC and organizational character (OC) (β = 0.545, *p* = 0.000) and the relationship between OC and TI (β = 0.236, *p* = 0.002) as well as the one between OC and BM (β = 0.295, *p* = 0.000) are all significant. Accordingly, H3 addresses some preliminary evidences. It requires further evidences by measuring the mediating effect of OC between IC and innovation.

**Table 5:** Standardized path coefficients.

**Path**	**Standardized coefficient**	***p*-value**	**Lower 2.5%**	**Upper 2.5%**
IC → OC	0.545^**^	0.000	0.467	0.825
IC → TI	0.264^**^	0.000	0.127	0.454
IC → BM	0.088	0.078	−0.012	0.231
OC → TI	0.236^**^	0.002	0.087	0.377
OC → BM	0.295^**^	0.000	0.217	0.411
TI → BM	0.310^**^	0.000	0.213	0.451

** *p < 0.01*.

**Figure 2:**
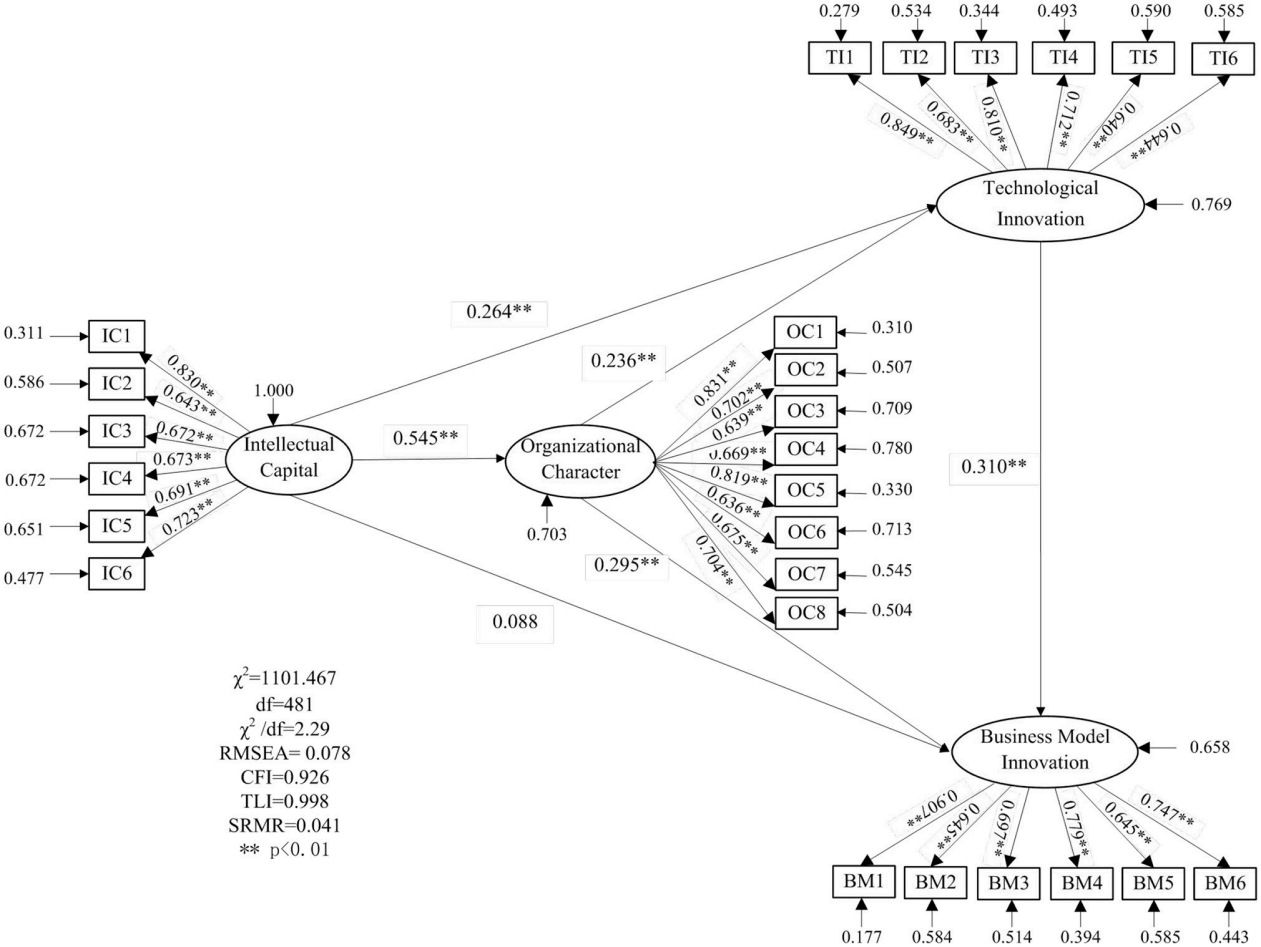
Effect paths among the variables.

The results of the path analysis are shown in Figure 2 in detail. As shown in Figure 2, the positive influence of IC on OC is very intense. Compared with TI, OC has a slightly stronger effect on BM. As for TI, the direct impact of IC is slightly stronger than the direct impact of OC. Moreover, TI is significantly related to BM.

### Mediating Effect

In the hypothesis design of this study, it only assumes that organizational character is a mediator. In fact, technological innovation can also be considered as a mediating variable which plays an intermediary role in the relationship between intellectual capital and business model innovation. The bootstrap program is used to test the mediating effects of organizational character and technological innovation, and the results are shown in Table 6.

**Table 6:** Coefficients of mediating effects.

**Path**	**Mediator**	**Standardized coefficient**	***p*-value**	**Lower 2.5%**	**Upper 2.5%**
IC → TI	OC	0.128^**^	0.005	0.059	0.284
IC → BM	OC	0.161^**^	0.000	0.126	0.297
	TI	0.082^**^	0.002	0.027	0.173
OC → BM	TI	0.073^**^	0.006	0.032	0.144

** *p < 0.01*.

As shown in Table 6, on the path from IC to TI, the mediating effect of OC is significant (β = 0.128, *p* = 0.005). That is, IC not only has a significant direct effect on TI, but also has a significant indirect effect through the partial mediation of OC. Therefore, H3 is supported. Respectively, on the path from IC to BM, OC plays a significant intermediary role (β = 0.161, *p* = 0.000), H3a is thus supported. The results that the mediating effect is significant while the direct effect is not significant indicate that OC plays a full mediating effect in the relationship between IC and BM. Hence, H3b is also supported. In addition, Table 6 shows that on the path from IC to BM, TI also plays a significant role as a mediator (β = 0.082, *p* = 0.002). Similarly, TI plays a significant mediating role in the acting path from OC to BM (β = 0.073, *p* = 0.006). The above results indicate that the indirect effects of IC on BM through OC and TI are all significant. OC and TI build a bridge from IC to BM.

When testing in bootstrap progress, the results also show that the direct effect of IC on TI is significant, with a 95% CI (confidence interval) not containing 0 ([0.127, 0.454]). IC is not significantly related with BM, with a 95% CI containing 0 ([−0.012, 0.231]). The bootstrapping results of the mediating effect indicate that the indirect effect of IC on TI through OC is significant, with a 95% CI not containing 0 ([0.059, 0.284]). Meanwhile, the bootstrapping results of the mediating effect indicate that the indirect effect of IC on BM through OC is significant, with a 95% CI not containing 0 ([0.126, 0.297]), and through TI is also significant, with a 95% CI not containing 0 ([0.027, 0.173]) because of TI's significant effect on BM, with a 95% CI not containing 0 ([0.213, 0.451]). In brief, bootstrap results confirm once again that IC has a direct role in promoting TI, but not in BM. That is, H1 is supported and H2 is rejected. OC plays an intermediary role in the impact of IC on TI as well as BM. Both H3a and H3b are supported, and H3 is therefore supported.

## Discussion and Implications

### Discussion

With the booming of the internet, digital economy, and e-commerce, China has ushered in a new era of economic development, namely, new economy. This concept was first officially incorporated into the national development strategy in the 2016 Government Work Report, China. According to the interpretation of Chinese Premier, Li Keqiang, the new economy covers the emerging industries which focus on the development of e-commerce and the internet economy as well as the upgrading traditional industries which focus on the applications of new technologies and new business models. From this perspective, it can be seen that the innovation centered on technological innovation and business model innovation provides the core driving force of the new economy. Under such circumstances, the exploration of the path to innovation has great significance in practice.

There may be a variety of path to innovation that has been developed (Adams et al., 2006). In the knowledge economy, many studies have demonstrated the relationship between intellectual capital and technological innovation (Subramaniam and Youndt, 2005; Delgado-Verde et al., 2016). It is believed that the interaction between technological innovation and knowledge production is a process of coupling (Leong et al., 2013; Lee, 2015). The accumulation of intellectual capital relies on knowledge production, while the former can also create conditions for technological innovation. The performance of technological innovation is manifested as the growth of knowledge. To some extent, China's new economy is an extension of the knowledge economy, but it also includes new connotations such as the new type of operation and new business model. Therefore, although intellectual capital has been proved to be the key antecedent of organizational innovation in the context of new economy, we are still required to seek new impetus to support the development of new type operations and new business models. In this research, we try to attribute the source of this new motivation to organizational character.

Organizational character includes both the psychological characteristics and behavior patterns of individual employees, as well as the psychology, preference, style, and pattern that an organization gradually establishes in the process of growth. Organizational character is actually the externalization result of intellectual capital after it is upgraded to the level of organizational mentality. Therefore, the accumulation of intellectual capital would be good for the formation and evolution of organizational character. Along with the creation and growth of intellectual capital, organizations would make relevant adjustments on strategic positioning, decision-making model, production process, and marketing channel, which would in turn imperceptibly change the mode of thinking and action, thus organizational character migrates. According to the research results of this study, intellectual capital expectedly has a significant impact on organizational character. The new contribution is that we find that the organizational character, which reflects the psychological trait and behavioral model of an organization, should be regarded as its mental center and has a significantly positive impact on both technological innovation and business model innovation. However, the impact of organizational character on technological innovation is different from its impact on business model innovation in terms of target, path or mechanism. Organizational character mainly influences the orientation, decision-making and implementation mode of technological innovation. Especially, the impact is concentratedly reflected in the early stage of technological innovation. Nevertheless, the influence of organizational character on business model innovation may run through the entire process of business model innovation including a series of activities such as strategic control, customer positioning, value construction and realization as well as profit model design. Moreover, the novelty creation of business model innovation depends on the heterogeneity of the organizational character, while the novelty creation of technological innovation depends more on the heterogeneity of intellectual capital. Therefore, the effect of organizational character on business model innovation would be slightly stronger than the one of organizational character on technology innovation. From these perspectives, our results can be well explained.

In several articles published in Chinese, the relationship between technological innovation and business model innovation has been demonstrated (Shuangmei and Rui, 2013; Mingming et al., 2014). To sum up, scholars think that there is a coupling and interactive relationship between them. In other words, the technological innovation and business model innovation are interdependent and mutually reinforced. Technological innovation provides technical support for business model innovation. Conversely, business model innovation provides technological innovation the way to achieve value. However, the action logic of an organization should follow the transformation process of knowledge-product-value, which starts from the goal of the organization and ends in the realization of corporate value. Therefore, the method that takes intellectual capital to promote technology and product innovation and then promotes value realization through business model innovation should be the inevitable choice of the organization's operational logic. The verified hypothesis that technological innovation positively promotes business model innovation can be interpreted and provide decision-making references for keeping the balance of technological innovation and business model innovation as well as making strategic arrangements for innovation.

To sum up, the path to innovation under the circumstance of China's new economy should be a path that starts from intellectual capital, bridges through organizational character, aims to promote technological innovation, and finally realizes the innovation of business model. In this path, business model innovation is the ultimate goal, and technological innovation is the tool and supporter; organizational character is the core driving force, while intellectual capital is the fundamental source of its motivation. Organizations can create a healthy, robust and sustainable innovation system by following this path, by which the driven transformation process of knowledge-product-value would generate continuous driving force for the value realization of organizations.

### Implications

#### Theoretical Implications

First, the enlightenment of this study for scholars is that we propose a new path to innovation in the new economy environment. This newly raised path has important theoretical significance for enriching the innovation theory. For one thing, we would remind more researchers to realize the significance of business model innovation and treat with the relationship between technological innovation and it. For another, we propose a totally new dual innovation theory which is different from the existing classification of dual innovations, e.g., exploratory innovation and exploitative innovation.

Second, we successfully introduce the organizational character into our research framework and prove its effective in explanation. It indicates a new perspective on observing the antecedent factors of innovation. In order to effectively promote innovation in the future, the intellectual capital and organizational character should be strengthened. The conclusion would open a new door for latecomers to study the path to innovation and the endogenous growth of organizations by leading them to explore the interactive relationships between intellectual capital and organizational character as well as to discuss the opportunity of collaborative governance. The collaborative governance of intellectual capital and organizational character may induce a new theory for managing intangible assets, which is different from the existing theory of intangible asset management.

#### Practical Implications

First, this study confirms that the intellectual capital has a direct or indirect effect on promoting technological innovation and business model innovation. This indicates that organizations should pay more attention to the development, accumulation and exploitation of intellectual capital through improving the business proficiency of employees, increasing systematic training to enhance their work skills, and developing social networks for knowledge sharing and business cooperation, etc. It is also recommended that organizations should communicate with stakeholders (especially the leading customers) frequently in order to perceive environmental and market changes timely and improve organizational adaptability and flexibility. In addition, organizations should scan and improve itself internally, for example, design efficient management processes, formulate sound management systems, or develop effective information technology platforms, in order to save organizational costs and improve operational efficiency.

In terms of the development of intellectual capital, Huawei's strategies can teach us a lot. Huawei has always spared no effort to travel all over the world and promised high salaries in order to obtain the best technical talents. It opened a university, Huawei University, for the training and exchange of its employees. Huawei has constructed an almost automated internal management system, by which the talents are no longer trapped in simple and repetitive works. In addition, Huawei provides a variety of communication channels for information exchange between itself and external environment, especially market, industrial technology and policy. The development of intellectual capital supports its successful innovation and makes it grow into a famous global enterprise with core competence.

Next, the results of this study reminds managers to pay more attention to the cultivation of organizational character. According to our study, organizational character plays a crucial role between intellectual capital and innovation; it can greatly promote the transformation of intellectual capital into business model innovation capability. Therefore, organizations should take notice of the shaping and cultivation of their characters. Through following the laws of the interaction and transformation of intellectual capital and organizational character to strengthen their combination in operations and facilitate the collaborative governance of them, organizations may continuously optimize and perfect their innovation paths and systems. Regarding to the cultivation of organizational character, organizations are required to make efforts on the internal cultivation and external presentation with assiduity. Internal cultivation covers the activities such as the creation of organizational culture and the establishment of employees' values and professional ethics. External presentation encourages such activities, for example, building a proper corporate image and brand awareness, assuming social responsibilities bravely, and making efforts on environmental protection, etc.

In regard to the management of organizational character, Uber is a typical case. In 2017, it was in crisis because of an article that revealed the company's culture, written by its former employee. According to the reveal, Uber's former CEO has created a value system which can be characterized by unscrupulous growth, neglected practice and trampled regulations, in order to realize rapid expansion. Cultural problems such as office harassment, office politics and male supremacy were serious in Uber. Uber's corporate image was been questioned until the departure of its founder and CEO. Dara Khosrowshahi, the new CEO of Uber, took a series of measures to reshape its internal culture and external image. He tried to identify its organizational character as decisiveness. On the one hand, he consulted the employees and re-written a set of corporate cultural norms. On the other hand, it began to actively shoulder social responsibility and respect regulatory agencies. After the reform, its order volume began to recover gradually.

In the end, this study also provides references for managers to treat with such issues, for example, how to carry on a new type of dual innovation. China's new economy has two distinctive features. The first one is the anabatic competition, since most of individuals have the opportunities to join the queue of developing new business and new economy. The second is the booming of new thought of development. In the circumstance of new economy, the fact that many small-scale business teams can achieve rapid development could attribute to their unique ideas that distinguishes them from others. In spite of the advantages of the accumulation of intellectual capital, large and medium-sized organizations should learn from some of small and micro organizations in terms of the latter's ability to develop business model innovation. Combining technological innovation with business model innovation is an effective way for organizations to survive and develop in the new economy environment. In order to answer the question how to organize (balance, match, combine and coordinate) this new dual innovation model, the study indicates that we can get the driving force for sustainable innovative development by centering the development and accumulation of intellectual capital and assisting with the cultivation and development of organizational character.

The comparative analysis of OPPO and Lenovo can provide more specific references for the development of dual innovation. In 2017, OPPO's mobile phone shipments (up to 120 million) ranked fourth in the world. By contrast, Lenovo's global shipments in 2017 were less than 30 million. As a long-established and large-scale corporation, Lenovo do have richer technological knowledge than OPPO. The relative backwardness can attribute to the difference of business model innovation capability. OPPO's sales miracle is supported by its particular business model that is compatible with customers and close to BOP market. Compared with OPPO's smartphone, Lenovo's featureless products do not have differentiated competitive advantages. It can be expected that OPPO would gain more space for development since it began to intensify its technological innovation based on the success of business model. In 2018, it creatively solved the problem of full-screen mobile phones. This may bring it a new round of growth.

## Conclusions, Limitations, and Future Research

### Conclusions

Based on a survey with 214 respondent questionnaires from Chinese corporate employees, this study explores the relationships among intellectual capital, organizational character, technological innovation, and business model innovation. The research results show us that the intellectual capital is the source of innovation and directly affects technological innovation. The organizational character bridges the influence path of the intellectual capital to innovation. It is positively affected by intellectual capital and then directly transfers the affection to technological innovation and business model innovation. Moreover, technological innovation has a significant role in promoting business model innovation. The mediating effects of organizational character and technological innovation is significant on the entire path. However, the direct impact of intellectual capital on business model innovation is not significant in our study.

According to the results, it can be concluded that organizations should take business model innovation and value realization as their ultimate goals. However, when they try to build or improve their innovation systems, they should not forget that the business model innovation needs to be supported by technological innovation. Other conclusions cover that the technological innovation depends on the support of intellectual capital and organizational character simultaneously, but the dependency of intellectual capital is slightly stronger than organizational character; business model innovation directly depends on organizational character, which also plays an indispensable intermediary role in the innovation system. Looking through the whole research framework we can find that the intellectual capital is the fundamental impetus source of innovation system.

According to the above conclusions, we make further inferences and propose the following three viewpoints. First, in the new economy environment, the innovation model of organizations should be a new type of dual innovation that integrates technological innovation and business model innovation. Only by coordinately managing the two at the same time and dealing with the mutual support and transformation relationship between them, can we create an effective and sustainable development innovation system. Second, in the new economy environment, organizations should make efforts on the collaborative governance of intellectual capital and organizational character. This new kind of management idea, which encourages to simultaneously develop and manage the intellectual capital and organizational character, would replace the knowledge management theory to provide better explanation for the future successful practices of organizations. Finally, in the new economy environment, the path to innovation in organizations is exactly a road from the co-governance of knowledge and character to dual innovation, but the influence mechanisms of intellectual capital and organizational character in the dual innovation system are different. We tend to explain it as an eccentrically conjugated mechanism with dual drivers. The core spirit of the mechanism is two-fold: first, the dual innovation must rely on the joint drive of intellectual capital and organizational character, that is, both of them are indispensable, and next, however, the functions of intellectual capital and organization character are compartmentalized and staggered in the driving force of technological innovation and business model innovation. More specifically, the intellectual capital drives more on technological innovation, while the organizational character pays more attention to the drive on business model innovation. The action that building a path to innovation in accordance with this mechanism becomes the key of organizational innovation in the new economy environment. Those above points are the core theoretical contributions of this study.

Compared with the existing theories, the study has its own advantages and disadvantages. In the literature, dual innovation research focuses on the ambidexterity between exploratory innovation and exploitative innovation, incremental innovation and disruptive innovation or other kinds of innovation, but almost all of them are limited in the field of technological innovation. This study elevates the position of business model innovation and proposes a new dual innovation system by integrating technological innovation and business model innovation. This would enrich the theory of dual innovation. The new proposition may certainly be challenged by some researchers who consider that the business model innovation is enclosed in the technological innovation since the former looks much like the behavior of commercialization and industrialization, which is one of sub-processes of the latter.

Furthermore, we were supposed to propose a new concept to define the combination of intellectual capital and organizational character, just like the human quotient theory including intelligence quotient (IQ) and emotion quotient (EQ). However, we give up this proposition ultimately because we worry that it would not be accepted easily by other researchers when the relationship and interactive mechanism between intellectual capital and organizational character are lack of deep exploration. A totally novel theory for the management of organization's immaterial factors, which is different from the intangible assets management theory, would be constructed from the perspective of the integration of intellectual capital and organizational character if we can provide more evidences in the future.

### Limitations and Future Research

Several limitations of our study should be examined further in future research. The first one is the problem of the sample. Although the sample size of this study has addressed the requirement of the structural equation model for parameter estimation (Fritz and Mackinnon, 2007), the sample size is actually insufficient compared to China's large population and number of companies. There may be some bias between the sample and the population. In future, it is necessary to expand the sample size to conduct more accurate measurement of relationships. However, it certainly would be a big project since the number of Chinese enterprise was up to 15 million in 2017, so we are difficult to effectively control the bias through sampling analysis under limited funding constraints. In any case, the results of this study are still valid and credible from the perspective of research methods about structural equation model and sampling statistics.

The followed one is the issue of cross-sectional research design. At the beginning, as we theoretically and practically delineate the causal relationships among the latent variables, we do not consider the potential reverse causalities. The cross-sectional design prevents us from deducing such causalities that differ from hypothetical relationships, although our results are consistent with our theoretical inferences. Accordingly, future research can focus on the collection of time-series data and the design of longitudinal research to reconstruct the model.

Finally, common method bias may affect our research results. Even though in this study the Harman's single factor test shows that common method bias is not a big problem, this problem does exist indeed. As demonstrated by Guide and Ketokivi (2015), all technologies have problems in dealing with common method biases, and there is no straightforward remedy for common method bias. Hence, future research should handle this problem by a more effective method from the beginning design.

## Ethics Statement

The data of this study were collected by questionnaire survey with the help of a professional service institute. The content of the questionnaire was not involved with any ethical problem. All respondents were informed of the aims of this research. It was indicated that they approved this study when they filled out the questionnaire. The authors obtained the consent of the participants when they completed the survey. In addition, an ethics approval was not required as the guidelines of the authors' institution, Nanchang University, and the regulations of the authors' country, China. For these reasons, the authors considered that we did not need to provide ethics approval and written informed consent.

## Author Contributions

DY designed and performed the research. JL processed the data and wrote the paper. All authors reviewed, edited, and approved the final manuscript.

### Conflict of Interest Statement

The authors declare that the research was conducted in the absence of any commercial or financial relationships that could be construed as a potential conflict of interest.
