# Supplementary material for: The Path to Innovation: The Antecedent Perspective of Intellectual Capital and Organizational Character
Source: Front Psychol. 2018 Dec 4;9:2445. doi: 10.3389/fpsyg.2018.02445 (PMC6290339; doi:10.3389/fpsyg.2018.02445)
Supplement: Supplementary file 1 [file Table_1.doc]

**Appendix**

**Table AI.** Variables and items

| Variables | | Items | Standard | References |
| --- | --- | --- | --- | --- |
| Latent Variable | Intellectual Capital  (IC) | (IC1) Most of our employees are highly skilled. | Five-point Likert scale: “1” means “strongly disagree”; “2” means “disagree”; “3” means “neutral”; “4” means “agree”; “5” means “strongly agree”. | (Subramaniam and Youndt, 2005) |
|  |  | (IC2) Our employees are creative and motivated. |  |  |
|  |  | (IC3) Our employees share information and learn from one another. |  |  |
|  |  | (IC4) Our employees are skilled at solving problems with customers, suppliers, or partners. |  |  |
|  |  | (IC5) Our organization uses manual, database, organizational system and workflow as a way to store knowledge. |  |  |
|  |  | (IC6) Our organization’s culture and value system (stories, rituals) contain valuable ideas, ways of doing business, etc. |  |  |
|  | Organizational Character  (OC) | (OC1) Our organization is very enthusiastic about both employees and customers. |  | (Chun and Davies, 2006) |
|  |  | (OC2) Our organization is based on integrity and undertakes social responsibility actively. |  |  |
|  |  | (OC3) Our organization keeps pace with the times and actively understands new trends in the industry. |  |  |
|  |  | (OC4) Our organization has a higher risk tolerance in expectations. |  |  |
|  |  | (OC5) Our organization is known for safety, reliability, and sense of responsibility. |  |  |
|  |  | (OC6) Our organization focuses on the achievement and is full of ideals. |  |  |
|  |  | (OC7) Our organization behave decently and is full of charm. |  |  |
|  |  | (OC8) Our organization has a good reputation and high prestige in the industry. |  |  |
|  | Technological Innovation  (TI) | (TI1) Our organization produces products that can replace old products on the market. |  | (Camisón and Villar-López, 2014) |
|  |  | (TI2) Our organization is able to extend the range of products. |  |  |
|  |  | (TI3) Our organization is able to improve product design continually. |  |  |
|  |  | (TI4) Our organization has developed a series of technological processes. |  |  |
|  |  | (TI5) Our organization master and absorb the basic and key technologies of business. |  |  |
|  |  | (TI6) Our organization continually develops programs to reduce production costs. |  |  |
|  | Business Model Innovation (BM) | (BM1) The business model offers new combinations of products, services and information. |  | (Zott and Amit, 2007) |
|  |  | (BM2) The business model brings together new and diverse participants. |  |  |
|  |  | (BM3) Our organization adopts novel approaches in its collaboration with partners. |  |  |
|  |  | (BM4) The business model gives access to an unprecedented variety and number of goods. |  |  |
|  |  | (BM5) The business model links participants to transactions in novel ways. |  |  |
|  |  | (BM6) Our organization has created new profit points. |  |  |
| Control Variable | Environmental Uncertainty (EU) | (EU1) The group of customer is still changing. |  | (Desarbo et al., 2005) |
|  |  | (EU2) Promotion approaches in the market emerge in an endless stream. |  |  |
|  | Hi-tech Enterprise Certification (HC) | Our organization has achieved high-tech enterprise certification. | 1. Yes; 2. No | According to China's customary semantics, Chinese statistical standards and enterprise managerial practices. |
|  | Ownership Structure  (OS) | The ownership structure of our organization is | 1. State-owned enterprise (SOE); 2. Foreign capital enterprise; 3. Joint-investment enterprise; 4. Private enterprise; 5. Other. |  |
|  | Staff Size  (SS) | The staff size of our organization is | 1. 10 or less (Micro firm); 2. 11-100 (Small firm); 3. 101-300 (Medium firm); 4. More than 300 (Large firm). |  |
|  | Age  (A) | The age of our organization is | 1. Less than 1 year; 2. 1-3 years; 3. 4-10 years; 4. More than 10 years. |  |
|  | Industry  (I) | Our organization is a member of | 1. Primary industry; 2. Secondary industry; 3. Tertiary industry. |  |
| Identity Item | Educational Background  (EB) | My educational background is | 1. Junior college or below; 2. Undergraduate degree; 3. Graduate degree. |  |
|  | Service Year  (SY) | I have worked in the organization for | 1. Less than 1 year; 2. 1-3 years; 3. 4-10 years; 4. More than 10 years. |  |
|  | Current Job  (CJ) | My current job is | 1. Manager; 2. Technician; 3. Production worker; 4. Salesman; 5. Other. |  |
